# The Utility of Gadoxetic Acid-Enhanced MR Imaging to Characterize Atypical Cirrhotic Nodules Detected on Dynamic CT Images

**DOI:** 10.1371/journal.pone.0107869

**Published:** 2014-10-13

**Authors:** Chen-Te Chou, Wen-Pei Wu, Chia-Bang Chen, Wei-Wen Su, Ran-Chou Chen, Yao-Li Chen

**Affiliations:** 1 Department of Biomedical Imaging and Radiological Science, National Yang-Ming Medical University, Taipei, Taiwan; 2 Department of Radiology, Chang-Hua Christian Hospital, Changhua, Taiwan; 3 Transplantation Medicine and Surgery Research Centre, Chang-Hua Christian Hospital, Changhua, Taiwan; 4 Department of Gastroenterology, Chang-Hua Christian Hospital, Changhua, Taiwan; 5 Department of Radiology, Taipei City Hospital, Taipei, Taiwan; 6 School of Medicine, Chung Shan Medical University, Taichung, Taiwan; 7 School of Medicine, Kaohsiung Medical University, Kaohsiung, Taiwan; University of Modena & Reggio Emilia, Italy

## Abstract

**Purpose:**

To evaluate whether gadoxetic acid (Gd-EOB-DTPA)-enhanced MR images of tumors taken during the hepatocyte-specific phase can aid in the differentiation between hepatocellular carcinoma (HCC) and dysplastic nodules (DNs) in patients with atypical cirrhotic nodules detected on dynamic CT images.

**Materials and Methods:**

Seventy-one patients with 112 nodules showing atypical dynamic enhancement on CT images underwent gadoxetic acid-enhanced MR imaging (MRI) studies. Using a reference standard, we determined that 33 of the nodules were DNs and that 79 were true HCCs. Tumor size, signal intensity on precontrast T1-weighted images (T1WI) and T2WI, and the pattern of dynamic enhancement on MR images taken in the hepatocyte-phase were determined.

**Results:**

There were significant differences in tumor size, hyperintensity on T2WI, hypointensity on T1WI, typical HCC enhancement pattern on dynamic MR images, or hypointensity on hepatocyte-phase images between DNs and HCC. The sensitivity and specificity were 60.8% and 87.9% for T2WI, 38.0% and 87.9% for T1WI, 17.7% and 100% for dynamic MR imaging, 83.5% and 84.9% for hepatocyte-phase imaging, and 60.8% and 87.9% for tumor size (threshold of 1.7 cm).

**Conclusion:**

Gd-EOB-DTPA-enhanced hepatocyte-phase imaging is recommended for patients at high risk of HCC who present with atypical lesions on dynamic CT images.

## Introduction

Hepatocellular carcinoma (HCC) is one of the most common malignant tumors and occurs mainly in patients with chronic liver disease, such as hepatitis B and C infections [1]. Although the 5-year survival rate of patients with HCC is low, early detection of HCC in cirrhotic livers markedly improves the survival rate [2].

According to the most recent recommendations by the American Association for the Study of Liver Diseases (AASLD), a diagnosis of HCC can be made if a mass larger than 1 cm shows features typical of HCC (hypervascularity in the arterial phase and washout in the venous/delayed phase) on contrast material-enhanced computed tomography (CT) or magnetic resonance (MR) imaging [3]. However, as many as 44% of HCCs show atypical features on dynamic contrast-enhanced images [4], [5], [6].

Gadoxetic acid is a liver-specific MR imaging contrast medium with combined perfusion and hepatocyte-selective properties [7], [8]. This agent has been demonstrated to increase the detection of focal liver lesions and to provide differential diagnostic information comparable to that provided by nonspecific extracellular gadolinium chelates [9]–[11]. To the best of our knowledge, the efficacy of gadoxetic acid in characterizing atypical cirrhotic nodules with atypical enhancement features on images derived from dynamic CT studies has yet to be properly elucidated. The purpose of this study was to evaluate whether gadoxetic acid-enhanced MR images of tumors taken during the hepatocyte-specific phase could differentiate between HCC and dysplastic nodules in patients with atypical cirrhotic nodules detected on dynamic CT images.

## Materials and Methods

### Patients

This study was approved by the institutional review board of the Changhua Christian Hospital and written informed consent was obtained from all of the patients who agreed to join the study. Patients with ultrasonographic evidence of focal liver tumors underwent dynamic CT studies for further lesion characterization. All of the patients had demonstrable risk factors for HCC, although none of them had a history of malignancy. The inclusion criteria for atypical cirrhotic nodules were as follows: a) tumor size > than 1.0 cm as measured on dynamic CT images; b) evidence of hypervascularity in the arterial phase without obvious contrast washout in the portovenous/equilibrium phase or isovascularity/hypovascularity in the arterial phase and hypodensity to adjacent liver parenchyma in the portovenous/equilibrium phase. In patients with more than three atypical cirrhotic nodules, only the largest three tumors were chosen for evaluation. During an 18-month period (Apr. 2011 to Oct. 2012), we prospectively enrolled 74 consecutive patients with focal liver tumors showing atypical enhancement on dynamic CT images. All of the patients underwent gadoxetic acid-enhanced MR imaging for further characterization of the focal lesions. Of them, three patients (3/74) were excluded from the study because they presented with other types of primary liver cancer (cholangiocarcinoma, n = 1; hepatocholangiocarcinoma, n = 2). Therefore, the final study population comprised 71 patients. The clinical characteristics of the 71 patients are shown in Table 1.

**Table 1:** Clinical characteristics of the 71 patients with hepatic nodules depitcing atypical AASLD HCC enhancement patterns during dynamic CT studies.

	Total patient number (n = 71)
Age (mean ± SD)	59.5±9.3
Gender	
Male	53
Female	18
Underlying liver disease	
HBV	36
HCV	22
HBV + HCV	2
Alcoholic	2
Cryptogenic	9
Child-Pugh class	
A	63
B	6
C	2
Alpha-fetoprotein	
normal (<20 ng/mL)	45
abnormal (≥20 ng/mL)	26

SD  =  standard deviation; HBV  =  hepatitis B virus; HVC  =  hepatitis C virus; HCC  =  hepatocellular carcinoma; AASLD  =  American Association for the Study of Liver Diseases.

Of the 71 patients, 4.2% (3/71) had multiple (>3) atypical cirrhotic nodules, 7.0%(5/71) had three atypical cirrhotic nodules, 35.2%(25/71) had two atypical cirrhotic nodules, and the remaining 53.5%(38/71) had one atypical cirrhotic nodule on dynamic CT studies. A total of 112 atypical cirrhotic nodules were included in the present study. The vast majority (85.7%, 96/112nodules were evaluated histopathologically. Of these nodules, 44.8% (43/96) were core needle biopsy specimens taken from 33 patients, 36.5% (35/96) were surgical specimens from 26 patients, and 18.8% (18/96) were lesions in explanted livers from 12 patients. The remaining 16 nodules (14.3%, 16/112),which were not examined histologically, were monitored either by CT or MR imaging at three-month intervals over a period of more than one year. None of the 16 nodules demonstrated changes in size or enhanced pattern during the follow-up periods and were considered DNs. Using a reference standard, we identified 33 DNs (mean size, 1.5±0.4 cm; range, 1.0–2.9 cm) and 79 HCC specimens (mean size, 2.1±0.9 cm; range, 1.0–3.1 cm), of which 35.4% (28/79) were well-differentiated (wHCC), 48.1% (38/79) were moderately differentiated (mHCC), and 16.5% (13/79) were poorly differentiated (pHCC).

### MR imaging

MR imaging of the liver was performed with a 1.5-T MR scanner (Magnetom Avanto, Siemens Healthcare, Erlangen, Germany) and a 16-channel body phased-array coil. Prior to administration of contrast material, axial breath-holding dual echo T1-weighted (T1W) spoiled gradient echo (GRE) imaging (TR/TE, 140 ms/2.3 ms and 4.4 ms; slice thickness, 5 mm, gap, 0.6 mm; matrix, 192×256; one signal acquired; flip angle, 70°; field of view [FOV], 36–40 cm), and breath-holding GRE T1W with fat saturation imaging (FS-T1WI) (TR/TE, 178–184 ms/2.4 ms; slice thickness, 5 mm, gap, 0.6 mm; matrix, 192×256; one signal acquired; flip angle, 70°; FOV, 36–40 cm) were performed. An integrated parallel acquisition technique (i-PAT) with an acceleration factor of 2 was applied to shorten the scan time and improve image quality. A BLADE technique was also applied to reduce motion artifacts.

For contrast-enhanced MR imaging, all patients received a 0.025 mmol/kg (0.1 ml/kg) dose of gadoxetic acid (Primovist, Bayer Schering Pharma, Berlin, Germany). A fluoroscopic bolus detection technique was used to determine the optimal timing for the hepatic arterial phase in all patients (CareBolus, Siemens Healthcare). The contrast agent was administered as a bolus at a speed rate of nearly 2 ml/sec through the peripheral veins. The line was then flushed with 20 ml of 0.9% saline. Dynamic three-dimensional T1 spoiled gradient-recalled echo sequence (volumetric interpolated breath-hold examination) imaging with chemically selective fat suppression (3D-T1WI) (TR/TE, 3.5–3.7 ms/1.4–1.6 ms; slice thickness, 3 mm; matrix, 192×256; one signal acquired; flip angle, 10°; FOV, 36–40 cm) was carried out before and arterial phase (mean, 12.9±1.3 s; range, 10 to 15 s), portal phase (mean, 42.6±1.4 s; range, 40 to 45 s and venous phase (mean, 72.6±1.4 s; range, 70 to 75 s) after the time of arrival of contrast material in the abdominal aorta. Equilibrium-phase FS-T1WI were obtained at 180 s using the same parameters as those used for precontrast FS-T1WI. T2-weighted turbo spin echo (TSE) imaging (T2WI, TR/TE, 3600–4000 ms/84 ms; slice thickness, 5 mm; gap, 0.6 mm; matrix, 320×320; echo train, 23; one signal acquired; flip angle, 140°; FOV, 36–40 cm) and T2WI with FS (FS-T2WI, 5000–5400 ms/90 ms; slice thickness, 5 mm, gap, 0.6 mm; matrix, 320×320; TSE factor, 20; one signal acquired; flip angle, 140°; FOV, 36–40 cm) were undertaken immediately after the equilibrium-phase FS-T1WI was completed [12]. A respiratory navigator was used to reduce respiratory motion artifacts. Twenty minutes after contrast agent injection, FS-T1WI and 3D T1WI were obtained using the same parameters as those used in the pre-contrast pulse sequences [13].

### CT imaging

**Figure 1:**
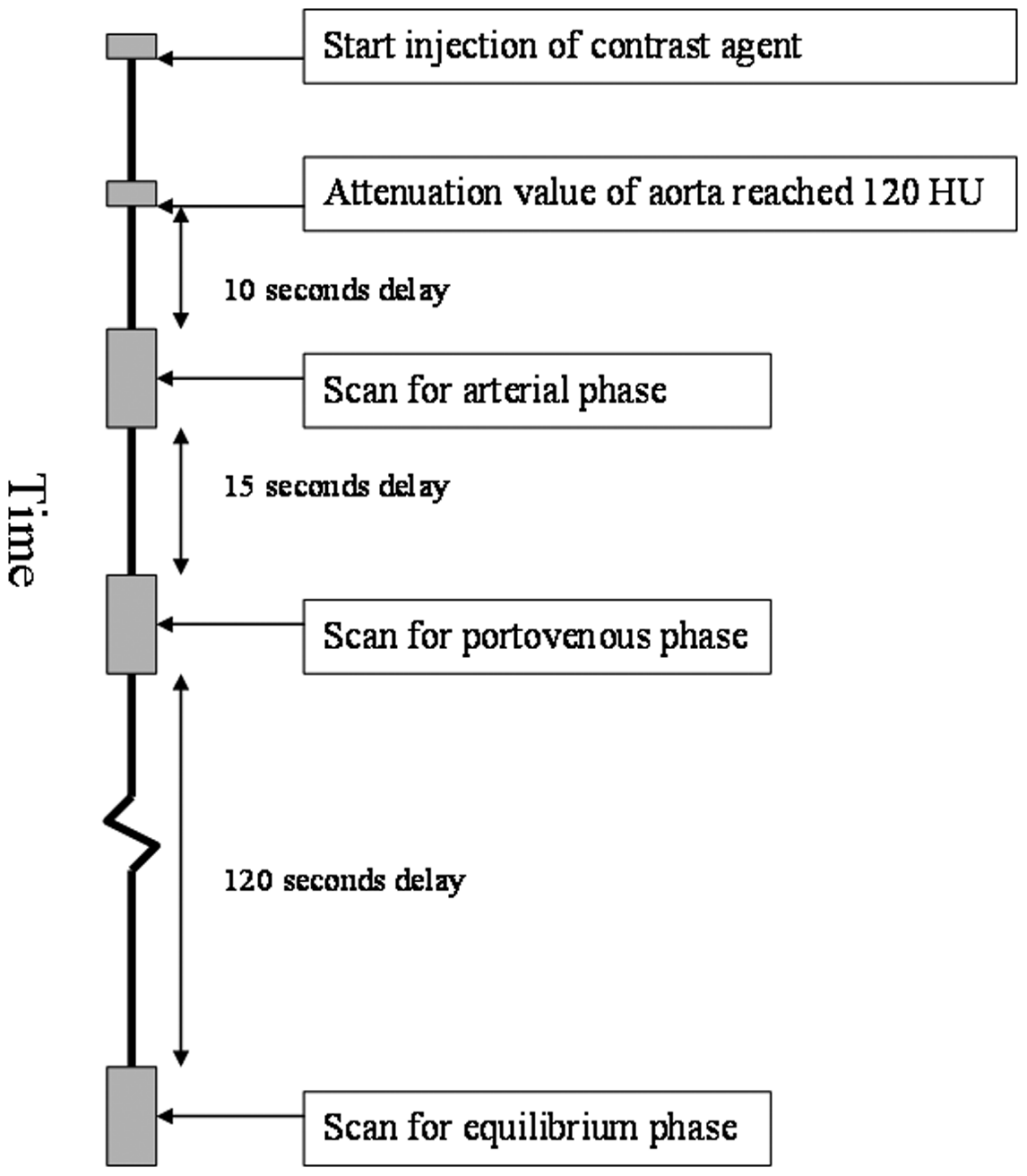
The imaging protocol for computed tomography dynamic study was shown.

CT images of the liver were obtained with a 16-slice multi-detector CT scanner (Lightspeed Ultra 16, GE Medical Systems, Milwaukee, WI, USA). Nonionic contrast medium (Omnipaque 350, General Electric Healthcare, Princeton, NJ, USA) was administered at a dosage of 1.5 mL/kg (mean total dose, 100 mL; range, 75 to 120 mL) with an injection rate of 3 mL/seconds through a 20-gauge venous cannula placed in the antecubital vein. For triphasic acquisitions, scanning was started with a 10-second scan delay for the hepatic arterial phase (mean, 27.0±1.3 seconds; range, 25 to 30 seconds, after injection of the contrast agent) after the attenuation value of the aorta reached 120 HU. Fifteen seconds after the end point of the hepatic arterial phase (mean, 52.1±1.2 seconds; range, 50 to 55 seconds), the scans for the portovenous phase were acquired. Equilibrium-phase images were acquired 120 seconds (mean, 172.1±1.2 seconds; range, 170 to 175 seconds) after the end of the acquisition of portovenous phase. Whole-liver scanning was completed in 4 to 8 seconds with the patients holding their breath. The CT scanning protocol is shown in Figure 1.

### Imaging evaluation

Imaging analysis was performed using a dual-screen diagnostic workstation (GE Healthcare, Milwaukee, WI, USA). In each image assessment, liver maps were created by drawing each individual liver lesion on a respective map, according to the Couinaud system of liver anatomy. This was done as accurately as possible by one author. All imaging results were independently analyzed using visual assessment by two radiologists who each had more than 10 years of experience in abdominal MR imaging. The two observers were blinded to the clinical information and final diagnosis, and they recorded the enhancement patterns on dynamic CT images, the signal intensity on pre-contrast T1WI/T2WI, the enhancement patterns on dynamic MR images, and signal intensity on hepatocyte-phase images. The signal intensity of the focal liver nodule on dual echo T1WI, T2WI, and hepatocyte-phase images was classified as hypointense, isointense, or hyperintense relative to the adjacent liver parenchyma. The presence of arterial enhancement of the lesion was detected by automatic subtraction using the software provided by the MR manufacturer. If a marked subtraction artifact due to position change was found, then manual evaluation of the changes in signal intensity of the lesion relative to those seen in the surrounding liver parenchyma for each phase of the dynamic study was done using an operator-defined region of interest (ROI). A circular ROI was drawn to encompass as much of the lesion as possible. The enhancement pattern of the HCC (relative to the adjacent liver parenchyma) was classified as hypovascular, isovascular, or hypervascular enhancement. Any difference of opinion between the two reviewers was resolved by a third radiologist who was also blinded to the clinical information and final diagnosis.

### Statistical analysis

Inter-observer differences between the initial two observers were evaluated with the Kappa statistic [14]. Continuous variables, such as age, tumor size, and alpha-fetoprotein level were analyzed by the Kruskal-Wallis test. Categorical variables, such as signal intensity and enhancement pattern, were analyzed by the Chi-square test. A receiver operating characteristic (ROC) analysis was performed to determine the size threshold needed to characterize the 112 atypical cirrhotic nodules seen on dynamic CT images. A *p*-value of less than 0.05 was considered to indicate statistical significance.

## Results

**Figure 2:**
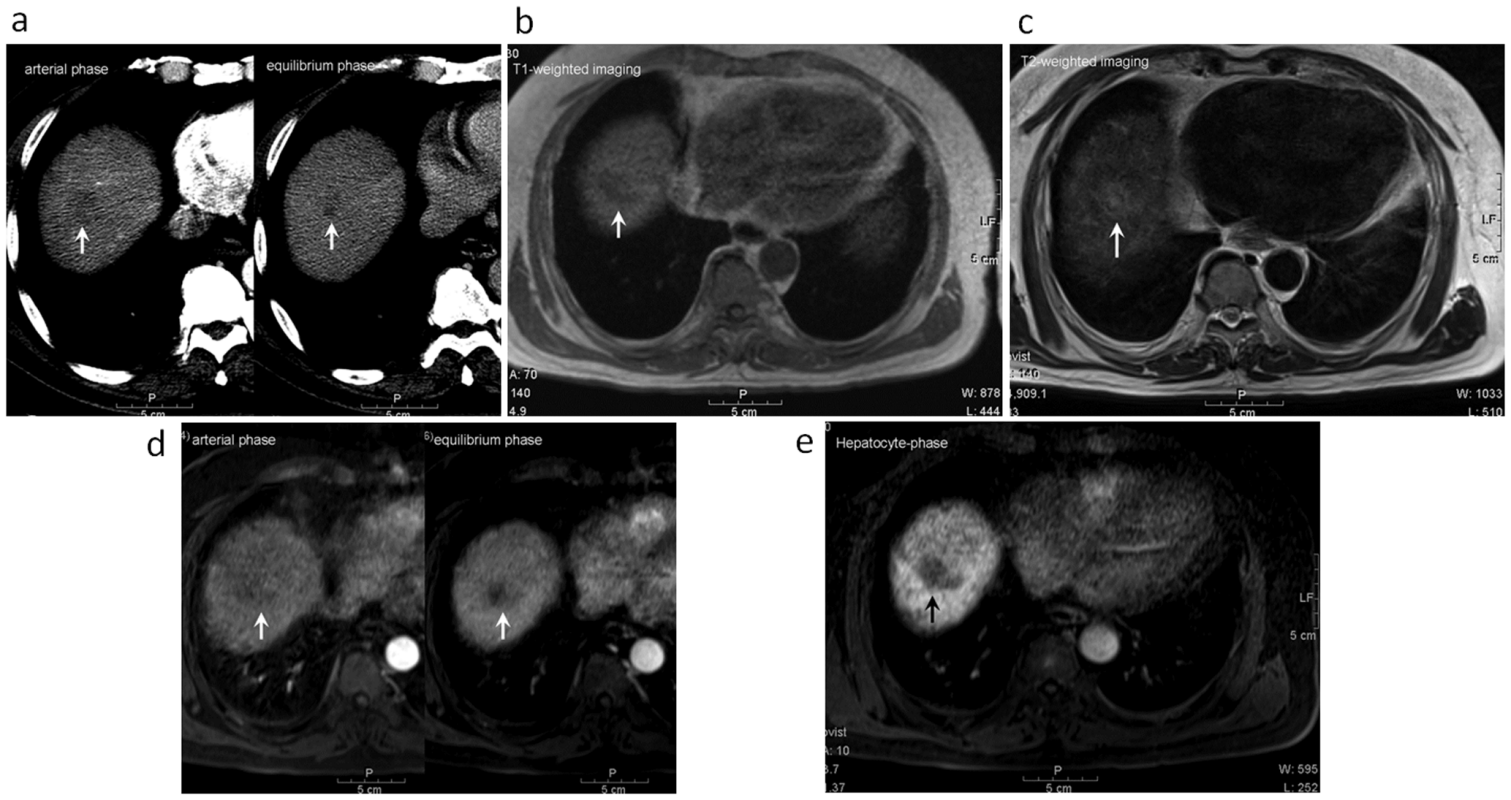
A 57-year-old male with a well-differentiated hepatocellular carcinoma (indicated by arrow) at S8 of the liver underwent gadoxetic acid-enhanced MR imaging and liver biopsy. (a) The tumor depicted hypovascular enhancement in the arterial-phase image and isodensity to hypodensity relative to the adjacent liver parenchyma on equilibrium-phase dynamic CT study. (b) The tumor showed hypointensity on the T1-weighted image. (c) The tumor showed isointensity to hyperintensity on T2-weighted image. (d) The tumor also showed hypovascular enhancement in arterial-phase imaging and hypointensity relative to the adjacent liver parenchyma on equilibrium phases of dynamic MR study. (e) The tumor showed hypointensity on the gadoxetic acid-enhanced hepatocyte-phase T1-weighted image.

**Figure 3:**
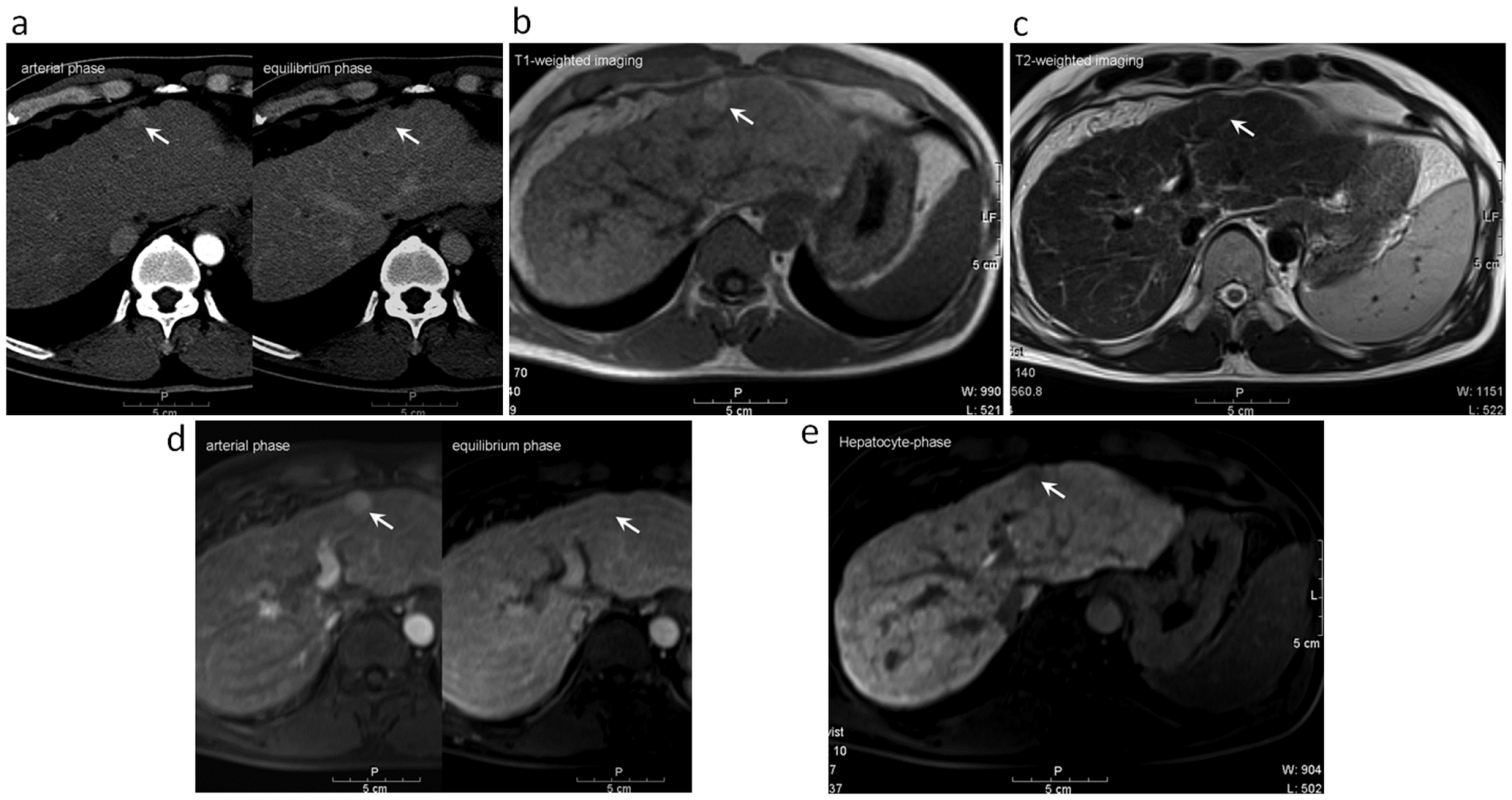
A 59-year-old male with a well-differentiated hepatocellular carcinoma (indicated by arrow) at S3 of the liver underwent gadoxetic acid-enhanced MR imaging and liver biopsy. (a) The tumor depicted hypervascular enhancement in the arterial-phase and isodensity relative to the adjacent liver parenchyma on the equilibrium phase of dynamic CT study. (b) The tumor showed hyperintensity on the T1-weighted image. (c) The tumor showed isointensity on the T2-weighted image. (d) The tumor also showed hypervascular enhancement in the arterial phase image and isointensity relative to adjacent liver parenchyma on equilibrium phases of the dynamic MR study. (e) The tumor showed hypointensity on the gadoxetic acid-enhanced hepatocyte-phase T1-weight image.

Excellent inter-observer agreement between the two reviewers was noted in the evaluation of the 112 atypical cirrhotic nodules seen on dynamic CT images and on gadoxetic acid-enhanced MR images (Table 2). Of the 112 atypical cirrhotic nodules, 77 showed hypovascularity/isovascularity in the arterial phase and were hypodense relative to the adjacent liver parenchyma in the venous phase (Figures 2a–e). The other 35 nodules showed hypervascularity in the arterial phase without washout phenomenon in the venous phase relative to the adjacent liver parenchyma on dynamic CT images (Figures 3a–e).

**Table 2:** The inter-observer difference for the study nodules on CT and MR imaging.

	Kappa value
MR imaging	
T2WI	0.881±0.061
T1WI	0.821±0.046
Fatty metamorphosis	0.846±0.045
Typical HCC enhancing pattern on MRI (AASLD)	0.868±0.064
Hepatocyte phase	0.906±0.041
CT imaging	
Arterial enhancement	0.858±0.052
Washout	0.837±0.052

T2WI  =  T2 weighted imaging; T1WI  =  T1 weighted imaging; AASLD  =  American Association for the Study of Liver Diseases.

As shown in Table 3, tumor size, T2W hyperintensity, T1W hypointensity, typical HCC enhancement patterns on dynamic MR images, and hypointensity on hepatocyte-phase images differed significantly between HCC lesions and dysplastic nodules. The area under the ROC, which was analyzed to measure the accuracy of tumor size in characterizing the 112 atypical cirrhotic nodules, was 0.749±0.047 (95% confidence interval: 0.658–0.862). The diagnostic accuracy rates of tumor size (threshold of 1.7 cm), hyperintensity on T2WI, hypointensity on T1WI, enhancement patterns on dynamic MR images, and hypointensity on hepatocyte-phase images are shown in Table 4. Only fourteen of the 79 HCC lesions with atypical enhancement patterns on CT images demonstrated enhancement features typical of HCC on gadoxetic acid-enhanced dynamic MR images. The other lesions (n = 65) had atypical enhancement patterns on dynamic MR images. The sensitivity of enhancement pattern as a diagnostic marker of HCC on dynamic MR images was only 17.7%. Of the 65 HCC lesions with atypical enhancement patterns on both CT and MR images, 52 showed hypointensity on hepatocyte-phase images. Only one nodule showed a typical HCC enhancement profile on dynamic MR images but hyperintensity on hepatocyte-phase images. However, five of the 33 DNs with hypointensity in hepatocyte-phase images were misdiagnosed as HCC. The value of hypervascularity on arterial-phase dynamic MR images plus hypointensity on hepatocyte-phase images as a diagnostic marker of HCC had a sensitivity of 46.8% and a specificity of 100%. The combination of hypervascularity on arterial-phase dynamic MR images and hypointensity on hepatocyte-phase images was found to be more sensitive than relying on enhancement patterns alone in dynamic MR images.

**Table 3:** MR characteristics of the 112 nodules showing atypical AASLD HCC enhancement patterns during CT dynamic studies.

MR imaging	HCC (n = 79)	DN (n = 33)	*P* value
Tumor size (cm)	2.1±0.9	1.5±0.4	<0.001
T2WI			
Hyper	48	4	<0.001
Iso/Hypo	31	29	
T1WI			0.012
Iso/Hyper	50	29	
Hypo	29	4	
Fatty metamorphosis			0.547
Yes	12	3	
No	67	30	
Typical HCC enhancing pattern on MRI			0.01
Yes	14	0	
No	65	33	
Hepatocyte phase			<0.001
Hypo	66	5	
Hyper-/Iso	13	28	
Hypervascularity on arterial phase of dynamic MRI			<0.001
Yes	41	5	
No	38	28	
Washout on venous phase of dynamic MRI			0.001
Yes	43	7	
No	36	26	

T2WI  =  T2 weighted imaging; T1WI  =  T1 weighted imaging; HCC  =  hepatocellular carcinoma; DN  =  dysplastic nodule; Hyper  =  hyperintense; Iso  =  isointense; Hypo  =  hypointense; AASLD  =  American Association for the Study of Liver Diseases; Value was depicted as mean ± standard deviation.

**Table 4:** The diagnostic performance of gadoxetic acid-enhanced MR imaging to differentiate between hepatocellular carcinoma (HCC) and dysplastic nodules.

MR imaging	Sensitivity	specificity	PPV	NPV	Accuracy
Tumor size (threshold of 1.7 cm)	59.5%	84.9%	89.8%	44.4%	67.0%
Hyperintensity on T2WI	60.8%	87.9%	92.3%	48.3%	68.8%
Hypointensity on T1WI	38.0%	87.9%	88.4%	37.2%	52.7%
Typical HCC enhancing pattern on MRI	17.7%	100%	100%	33.7%	41.1%
Hypointensity on hepatocyte-phase	83.5%	84.9%	93.0%	68.3	83.9
Hypervascularity on arterial phase plus hypointensity on hepatocyte-phase	46.8%	100%	100%	44%	62.5%

T2WI  =  T2-weighted imaging; T1WI  =  T1-weighted imaging; PPV  =  Positive predictive value; NPV  =  Negative predictive value.

Hyperintensity or isointensity on gadoxetic acid-enhanced hepatocyte-phase images was noted in 36% (9 of 28) of the well-differentiated HCC specimens and in 11% (4 of 38) of the moderately differentiated specimens. A total of 16.5% (13 of 79) HCC specimens with atypical enhancement patterns on CT images demonstrated atypical features on gadoxetic acid-enhanced hepatocyte-phase images.

## Discussion

Small HCC lesions, defined as hepatic lesions measuring less than 2 cm in diameter, frequently present with atypical features in dynamic studies, making them challenging to diagnose in daily practice [15], [16]. It is important to differentiate between benign cirrhotic nodules and atypical HCC because treatment of the latter differs markedly from treatment of the former [2], [17]. According to AASLD practice guidelines, patients with nodules ≥1 cm in diameter depicting atypical enhancement profiles in dynamic CT studies should undergo dynamic MR studies to further characterize the hepatic lesions [3]. In this study, we followed the AASLD guidelines and found that repeated MR dynamic studies enabled us to correctly characterize only 17.7% (14 of 79) of the HCC lesions with atypical enhancement patterns seen on CT images. We found that the combination of hypervascularity seen on images taken during the arterial phase and hypointensity seen on images taken during the hepatocyte phase of dynamic MR imaging provided higher sensitivity (46.8%) than but the same specificity (100%) as that of typical enhancement patterns seen on dynamic CT images in characterizing atypical cirrhotic nodules. According to our results, we recommend that clinicians use gadoxetic acid-enhanced MR studies in combination with hepatocyte-phase imaging for patients in whom dynamic CT studies disclose atypical enhancement of hepatic lesions.

We found that hyperintensity on T2-weighted images could be used to differentiate between DNs and true HCC lesions in patients with atypical enhancement patterns on CT images. Hyperintensity in T2-weighted images is an independent and strong risk factor at baseline for subsequent hypervascularization in hypovascular nodules in patients with chronic liver disease [18]. Ouedraogo et al. also reported that adding T2-W hyperintensity to the AASLD criteria increases the detection of HCC, especially for nodules smaller than 20 mm [19]. According to our results, hyperintensity in T2-weighted images offers additional information that can be used to differentiate between true HCC and DNs in patients with CT images of hepatic nodules showing atypical enhancement patterns. Therefore, we recommend that biopsy specimens of cirrhotic nodules showing hyperintensity on T2-weighted images be taken.

In our study, 16.5% (13 of 79) of the HCC lesions showed atypical presentations (hyperintensity or isointensity) on images taken during the hepatocyte-phase. This rate was much higher than that reported previously (5% to 10%) [20], [21]. The higher rate reported in this study might be due to the fact that a third (28 of 79) of the HCC lesions in our study were well-differentiated HCC. Well-differentiated HCC lesions tend to show higher contrast enhancement in hepatocyte-phase imaging [22]. In our study, 35.7% of well-differentiated HCC specimens showed atypical patterns on hepatocyte-phase images, a percentage which is similar to that reported by Kawada et al. [22].

We found that hypointensity on T1-weighted images and tumor size were also significant predictors of true HCC. Our results were similar to those reported in previous studies, which showed that the majority of dysplastic nodules were hyperintense/isointense on T1-weighted images and that the majority of true HCC lesions were hypointense on T1-weighted images [23]. Previous studies have demonstrated that larger tumor size is indicative of higher histopathologic grade [24], [25]. Eguchi et al. reported that the mean size (15.8 mm) of dysplastic nodules containing cancerous foci was significantly larger than the mean size (10.1 mm) of other dysplastic nodules [26]. Based on our results, atypical cirrhotic nodules larger than 1.7 cm on dynamic CT images should be investigated aggressively (e.g. biopsy or follow-up at short interval).

This study had several limitations. First, when patients had multiple nodules within the cirrhotic liver, only the largest three nodules were included. Further investigation of nodules in explanted livers is necessary to validate all cirrhotic nodules. Second, not all atypical cirrhotic nodules were investigated histologically. This is a known shortcoming in most comparative studies [27]. However, histological confirmation of each atypical cirrhotic nodule for patients with multiple nodules is time consuming and not very realistic in clinical practice. Therefore, the diagnosis was based on long-term CT or MR follow-up studies (≥1 year). Third, there was an imbalance in the number of benign and malignant lesions (33 DNs vs. 79 HCCs) in our study. This is because we only included nodules larger than 1.0 cm that were visible on CT images. However, studies have shown that the majority of dysplastic nodules are smaller than 1.0 cm and are isodense to liver parenchyma on CT studies [28].

In conclusion, the combination of hypervascularity on arterial-phase and gadoxetic acid-enhanced hepatocyte-phase T1-weighted images provides additional information that can be used to characterize cirrhotic nodules with atypical enhancement patterns on images taken during dynamic CT studies. Gadoxetic acid-enhanced MR studies with hepatocyte-phase imaging instead of conventional gadolinium-enhanced MR studies are recommended for patients with atypical cirrhotic nodules seen on images taken during dynamic CT studies.
